# Shugan Yiyang capsule for the treatment of erectile dysfunction: A protocol for systematic review and meta-analysis of randomized controlled trials

**DOI:** 10.1097/MD.0000000000017646

**Published:** 2019-11-01

**Authors:** Xuhong Yan, Junjun Li, Fang Yang, Xiaopeng Huang, Kun Tan, Liang Dong, Xujun Yu

**Affiliations:** a Hospital of Chengdu University of Traditional Chinese Medicine; b Department of Andrology, The Reproductive and Women-Children Hospital, Chengdu University of Traditional Chinese Medicine; c Chengdu University of Traditional Chinese Medicine, Chengdu, Sichuan, P.R. China.

**Keywords:** erectile dysfunction, IIEF-5, PDE5-I, Shugan Yiyang capsule, systematic review

## Abstract

**Background:**

Erectile dysfunction (ED) is a common disease. It affects the quality of life of both husband and wife and becomes an independent risk factor for cardiovascular events. In China, Shugan Yiyang (SGYY) capsule has been increasingly reported in clinical trials for the treatment of ED and reported inconsistent findings. Therefore, it is necessary to conduct a systematic review to evaluate the efficacy and safety of this drug for the treatment of ED.

**Methods:**

Chinese and English literature of SGYY capsule for ED published before August 31, 2019 will be comprehensive searched in PubMed, Cochrane Library, EMBASE, WANFANG, China National Knowledge Infrastructure, VIP Chinese Science and Technology Journal Database, Chinese biomedical document service system, and Clinicaltrials.gov. All randomized controlled trials that meet the eligibility criteria will be included and other studies will be excluded. Two investigators will conduct literature screening, data extraction and assess risk of bias alone, and the third investigator will handle disagreements. Two outcomes involving the international index of erectile function 5 score and adverse events will be evaluated. RevMan 5.3 and Stata 14.0 will be used to conduct this systematic review. The preferred reporting items for systematic reviews and meta-analysis protocols (PRISMA-P) statement is followed in this protocol and the the PRISMA statement will be followed in the completed systematic review.

**Conclusion and dissemination:**

The efficacy and safety of SGYY capsule for ED will be evaluated. We will publish the results of this systematic review in peer-reviewed journals to provide new evidence to clinicians.

Registration information: PROSPERO CRD42019140903

## 1 Introduction

Erectile dysfunction (ED) refers to the inability of men to obtain and maintain adequate penile erection to maintain satisfactory sexual intercourse, and it is one of the most common male sexual dysfunctions.^[1]^ It's global prevalence reached 3% to 76.5%^[2]^ and the prevalence among young men reached 30%.^[3]^

ED has a negative impact on both spouses^[4]^ and is positively associated with cardiovascular disease^[2]^ and has become an independent risk factor for predicting cardiovascular events.^[5]^ The first-line drug treatment for ED is phosphodiesterase type 5 inhibitors (PDE5-Is), but still some patients have little effect with them, and various side effects existed.^[6]^ In China, besides the use of western medicine such as PDE5-Is, patients and doctors also prefer to choose traditional Chinese medicine (TCM) for treatment.^[6,7]^ It has been reported that the combination of TCM and PDE5-I was better,^[8,9]^ and the adverse reactions were less than that of using the PDE5-I alone.^[9]^

Based on TCM theory, it is believed that the pathogenesis of ED is closely related to “liver,” “kidney,” and “blood stasis.”^[7,8]^ The Chinese patent medicine Shugan Yiyang (SGYY) capsule was developed by this theory. A multicenter, randomized, double-blind, controlled trial showed that it improved ED and was superior to placebo and positive control Chinese patent medicine, with no significant adverse effects.^[10]^ Animal experiments showed that SGYY capsule improved ED of rats by regulating mediators of nitric oxide synthase-cyclic guanosine monophosphate pathway.^[11]^ It also significantly upregulated vascular endothelial growth factor, insulin-like growth factor, protein kinase B and improved vascular endothelial function thereby improving erectile function of rats.^[12]^

In recent years, the clinical trials of SGYY capsule have increased, but there are inconsistent results.^[10,13]^ This brings confusion to the clinical application for clinicians. Therefore, it is necessary to carry out a systematic review and meta-analysis to fully evaluate the efficacy and safety of SGYY capsule in the treatment of ED.

## 2 Review objectives

The purpose of this systematic review is to evaluate the efficacy and safety of SGYY capsule for the treatment of ED, provide evidence-based medical evidence and suggestion for further research in the future.

## 3 Methods

This is a systematic review, and the meta-analysis will be carried out as conditions permit. Since this is a systematic review based on original research, no ethics committee approval is required.

### 3.1 Protocol and registration

Authors have completed the registration of the study on Prospero. Registration number: CRD42019140903

The preferred reporting entries of the PRISMA statement for system review and meta-analysis protocols (PRISMA-P)^[14,15]^ will be followed in this protocol. And the PRISMA statement will be followed when report the systematic review.

### 3.2 Data source

#### 3.2.1 Electronic search approach and database

We will systematically search English literature in Cochrane library, EMBASE, PubMed, and Chinese literature in China National Knowledge Infrastructure (CNKI), Chinese biomedical document service system (SinoMed), VIP Chinese Science and Technology Journal Database (VIP), WANFANG data. The literature publication deadline is August 31, 2019 in each platform or database and the search work will be done in September, 2019. The literature search update will be executed again before the systematic review is completed.

Subject heading, free text words will be used to search in Cochrane library, EMBASE, PubMed. In Cochrane library and EMBASE, the using of free words will be limited within title, abstract and keywords, but in PubMed, limited in tittle/abstract. The “topic” field will be used for the search of CNKI and WANFANG, and the “title or keyword” filed for the search of VIP. The subject heading plus free words form will be used to retrieve SinoMed.

We will use search terms involving “Erectile dysfunction” or “impotence” or “ED” AND “Shugan Yiyang” or “Shuganyiyang.” The Chinese form of the above words will be searched in Chinese databases. The specific search strategies of PubMed are shown in Table 1.

**Table 1:**
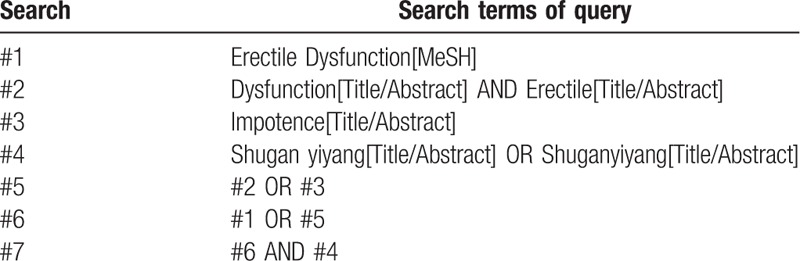
PubMed search strategies.

#### 3.2.2 Other sources of search

We will search Clinicaltrials.gov for registered clinical trials. Baidu's academic search engine and library interlibrary loan will be used to assist in full-text access or literature access.

### 3.3 Included and excluded criteria

#### 3.3.1 Study design

Randomized controlled trials (RCTs) that meet the eligibility criteria will be included. Other type of studies will be excluded including case reports, patient series, retrospective studies, self-controlled or before and after controlled studies, animal studies, reviews, laboratory researches, observational studies.

#### 3.3.2 Participants.

##### 3.3.2.1 Included population

ED patients with an international index of erectile function 5 (IIEF-5) score less than 22 will be included. ED defined as the inability of men to obtain and maintain adequate penile erection to maintain satisfactory sexual intercourse.^[1]^ No limit with age, severity, primary or not and accompanying diseases.

##### 3.3.2.2 Excluded population

ED caused by the following will be ruled out: vascular diseases include venous leaks, arteriosclerosis; neurogenic factors such as central nervous system injury, spinal cord injury; anatomical origin such as abnormal penile structure; injury; surgery; the original study included patients with severe organic or functional lesions of heart, liver, kidney, or patients with poor glycemic control of diabetes.

#### 3.3.3 Interventions

All RCTs which contrast SGYY capsule with other drugs or placebo will be included whether SGYY capsule is an intervention or control measure. If SGYY capsule is used as a control in the trial and another drug is an intervention, we consider reversing the order of the 2 interventions in this systematic review, that is, SGYY capsule will be regarded as an intervention measure, and the other drug as a control measure. Limited to RCTs for drug therapy. Drug therapy in intervention group defined as SGYY capsule, SGYY capsule combine with Western medicine. We will exclude studies of combined nondrug therapies in interventions such as physical therapy, surgical therapy, and so on.

#### 3.3.4 Control measures

Placebo, Western medicine, or other Chinese patent medicine as control measure will be included. The control measure can be 1 drug or combination of more than 1 drug. We will exclude non-Chinese patent medicine involving ointment, decoction, and other nondrug therapy including acupuncture, moxibustion, and so on.

#### 3.3.5 Outcomes

##### 3.3.5.1 Primary outcome indicator

IIEF-5 score.

##### 3.3.5.2 Secondary outcome indicator

Adverse events: all adverse events reported in the included studies.

### 3.4 Literature screening

**Figure 1:**
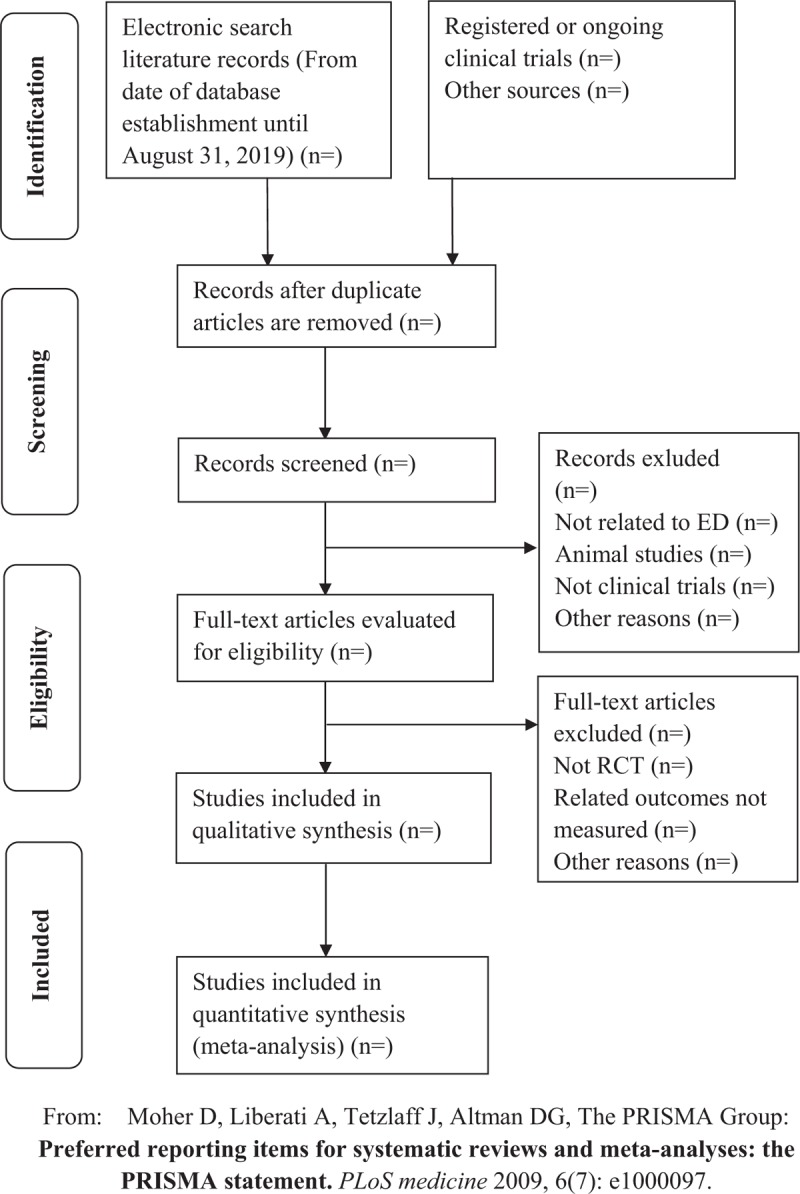
The PRISMA flow chart. PRISMA = preferred reporting items for systematic reviews and meta-analysis.

We will use endnote X8 software for document screening and document management. First, repeated literature screening will be implemented. The preliminary judgment basis information of the repeated literature includes title, author, abstract, keyword, journal, page number. Second, duplicate documents will be removed by reading the title and abstract. If there are similar or even same information both in the abstract of the conference paper and the full-text article, such as the author, research design and implementation, research results data, and so on, we will consider them to be the same research and only the full-text article will be included. We will include the study with the largest sample size and the most comprehensive data in case some original studies with the same samples or participants, such as a subcenter study and its whole multicenter study results, or different data results on different time in the same study. When the team member is unable to judge the duplication, the original research author will be contacted by email to assist in the judgment. Two authors (Xuhong Yan, Junjun Li) will conduct the literature screening process independently, and the controversial areas will be resolved through discussions with another member (Fang Yang). The flow chart of planned literature screening is shown in Figure 1.

### 3.5 Data extraction

The group will discuss the information to be extracted and make a data extraction form. Ahead of that, exercises will be performed by 2 authors alone. The data in 3 to 4 articles will be extracted to verify the consistency, comprehensiveness, and accuracy. The formal data extraction process will be carried out independently by 2 systematic review authors (Xuhong Yan, Junjun Li), and all disagreements will be discussed with the third member (Fang Yang). If there is any dispute that cannot be resolved by the team members, the original research author will be contacted by email to help. The following information will be extracted from the original studies.

(1) General information of studies: the 1st author, year of publish, article title, research location, conduct time, corresponding author, and e-mail.
(2) Design and methodology of studies: design type, sample size, information of randomize, allocation concealment, blindness information, diagnostic criteria, outcome indicators and measurements, safety indicators, methods and time of follow-up, statistical methods.
(3) Participants/patients: age, ethnic group, severity of disease, course of disease, baseline level, diagnosis of disease, syndrome differentiation of TCM, comorbidity.
(4) Information of intervention and control: name of drug, dosage, frequency, method of administration, duration, combined medication and therapy.
(5) Data of outcome indicators: the IIEF-5 score measured at different time points; adverse events number, and specific information.
(6) Data for assessing risk of bias: random sequence generation, assignment hiding, blind to patients and researchers, blind measurement, data integrity, selective reporting, other bias.
(7) Other data: conflict of interest, source of funding.

### 3.6 Risk of bias assessment

The risk assessment of the bias will be independently taken by 2 reviewers (Xuhong Yan, Junjun Li) based on the extracted data information. Any inconsistencies will be discussed and resolved with the third author (Fang Yang). This process will be based on the Cochrane Collaboration's tool for assessing risk of bias.^[16]^ Assessment items according to the information of random sequence generation, assignment hiding, blind to patients and researchers and blind measurement, data integrity, selective reporting, other bias.^[16]^ The results of the assessment will be shown as high risk, unclear, and low risk.^[16]^ The outcome of the assessment of risk of bias will be presented in tabular form or a specific figure made by using Review Manager 5.3 software.

### 3.7 Data analysis and synthesis

If the clinical heterogeneity between the included clinical trials is significant, or the data from the original study cannot be extracted, we will perform descriptive analysis or narrative synthesis. Only when the apparent clinical heterogeneity between studies is excluded and the data are sufficiently similar and homogeneous, the meta-analysis is conducted.^[17,18]^ Chi-square test will be used to test the heterogeneity and *I*^*2*^ statistic will be used to test the size of heterogeneity.^[17,18]^ There is heterogeneity when the *P*-value of the Chi-square test ≤.10, but no heterogeneity while the Chi-square test *P*-value > .10.^[17,18]^ We define *I*^*2*^ ≤ 50% for acceptable heterogeneity in multiple studies.^[17,18]^ In this case, the fixed model will be applied to calculate mean differences (MDs) by inverse variance and risk ratios (RRs) by Mantel–Haenszel method.^[17,18]^ When *I*^*2*^ > 50%, high heterogeneity between studies is considered. In this case, the causes of heterogeneity such as the age, the severity of the condition, the dose and the length of the intervention will be analyzed and subgroup analysis will be used.^[17,18]^ If there still have higher heterogeneity after the above methods processed, random model will be conducted in meta-analysis.^[17]^

MDs and 95% confidence intervals (CIs) will be used for the effect size of the numerical variable, and RRs and 95% CIs for the effect size of dichotomous variable.^[17,18]^ The effect size will be measured by *Z* test, and the *P-*value ≤.05 is statistically significant.^[17,18]^ The results of the meta-analysis will be presented as forest plots by RevMan 5.3.

### 3.8 Subgroup analysis

Subgroup analysis will be performed according to age, the severity of disease, the different dose and administration of interventions, different time point of outcome measurement.

### 3.9 Sensitivity analysis

We will use sensitivity analysis to test the stability and reliability of meta-analysis. It will be conducted by 2 methods: eliminating each study one by one; using random-effect model (DerSimonian & Laird method) to test the results after using the fixed effect model.^[17,18]^

### 3.10 Publication bias

Egger test (by Stata software 14.0) will be used to test publication bias and funnel plot (by Review Manager 5.3) be used in case the number of included trials reaches 10.^[17,18]^

## 4 Discussion

In China, a large number of patients choose Chinese medicine to treat diseases every year. TCM medical institutions served 1.02 billion patients in 2017, reached 15.9% of the total medical service in China.^[19]^ Therefore, providing evidence-based medical evidence of Chinese medicine is conducive to serving patients.

ED is a common disease but not adequately treated over the world.^[20]^ Although current literature recommends PED5-I regulators, vacuum erection devices, intraurethral and intracavernosal injection therapies, and penile prosthesis,^[1,7,20,21]^ Chinese medicine is still a choice for ED patients in China and all over the world.

“Yang Wei” is the name of ED in Chinese medicine, and there are a lot of discussions and clinical experiences concerning this disease.^[6,7]^ TCM can improve systemic symptoms and is mainly used for psychological ED, mild or moderate organic ED.^[6]^ Some Chinese herbs have androgenic effects, which can improve sexual desire.^[6]^

Psychological distress belongs to the category of liver depression in TCM,^[7]^ and it interact with ED.^[20]^ The pathogenic mechanism of ED in TCM includes liver depression, kidney deficiency, blood stasis, and meridian block.^[7,8]^ The above factors interact to collectively lead to ED.^[7]^ In addition, patients with kidney deficiency can occur with loss of libido, lack of energy, or fatigue.^[22]^ In the theory of TCM, SGYY capsule has the functions of soothing liver and relieving depression, promoting blood circulation and tonifying kidney.^[10]^ A study shows that SGYY capsule can improve systemic symptoms such as anxiety, depression, fatigue, and impotence in patients with chronic fatigue syndrome.^[22]^ The recommended treatments in the current Chinese guidelines for ED include the combines of PDE5-Is, SGYY capsule, and psychological counselling.^[8]^ There are some evidences based on RCTs for the treatment of ED with SGYY capsule, but no relevant systematic review. Therefore, we will make a systematic review to provide evidence-based medical evidence for the clinical use of SGYY capsule. It will also provide recommendations for further researches in the future.

This systematic review uses the IIEF-5 questionnaire score as an outcome indicator. The questionnaire has 5 questions, including erection confidence, erection hardness, maintenance of erection, persistence of erection, and the satisfaction of sexual intercourse.^[6,7,23]^ The IIEF-5 score (a total of 25 points) is divided into normal erectile function (≥22 points); mild ED (12–21 points); moderate ED (8–11 points); severe ED (<8 points).^[6,7]^ Although this questionnaire is subjective, it has been verified, generally acknowledged and has become an important tool for evaluating ED.^[23–26]^

We recognize that this study has some limitations. First, there may not be enough large samples of RCTs. Second, the quality of some RCTs may not be high and will affect the overall quality of the evidence. Therefore, we hope there will be more large-scale, multicenter, high-quality RCTs providing high-quality evidence in the future.

## Author contributions

**Conceptualization:** Xuhong Yan, Junjun Li, Xujun Yu.

**Data curation:** Xuhong Yan, Junjun Li, Fang Yang.

**Formal analysis:** Xuhong Yan, Junjun Li, Kun Tan, Liang Dong.

**Funding acquisition:** Xiaopeng Huang, Xujun Yu.

**Investigation:** Xuhong Yan, Junjun Li, Fang Yang, Xujun Yu.

**Methodology:** Xuhong Yan, Kun Tan, Liang Dong.

**Project administration:** Xuhong Yan, Junjun Li, Xiaopeng Huang, Xujun Yu.

**Resources:** Xiaopeng Huang, Xujun Yu.

**Software:** Xuhong Yan, Junjun Li, Fang Yang, Kun Tan.

**Supervision:** Xuhong Yan, Junjun Li, Xiaopeng Huang, Kun Tan, Xujun Yu.

**Validation:** Xuhong Yan, Fang Yang, Liang Dong, Xujun Yu.

**Writing – original draft:** Xuhong Yan, Junjun Li.

**Writing – review and editing:** Fang Yang, Xiaopeng Huang, Kun Tan, Liang Dong, Xujun Yu.
